# Gas in Your Stomach? A Curious Case of Complicated Emphysematous Gastritis With Concomitant Portal Venous Gas and Pneumoperitoneum Caused by Candida Glabrata

**DOI:** 10.7759/cureus.11650

**Published:** 2020-11-23

**Authors:** Eric Landa, Erika Vigandt, Ying Zhu, Ismail Ganim

**Affiliations:** 1 Internal Medicine, Unity Health, Searcy, USA; 2 Internal Medicine, Brooklyn Hospital Medical Center, Brooklyn, USA

**Keywords:** gastritis, emphysematous gastritis, gastrectomy, pneumoperitoneum

## Abstract

Emphysematous gastritis is a rare life-threatening infection caused by gas trapping within the gastric mucosal wall. It is diagnosed by radiological or operative findings most typically by CT scan of the abdomen. It is caused by gas-producing bacteria. Predisposing factors include but are not limited to alcohol intake, trauma, diabetes and surgery. Clinical presentation will typically include severe abdominal pain, abdominal distension and shock. Here we present the only reported case to our knowledge of Emphysematous gastritis with concomitant portal venous gas and pneumoperitoneum caused by Candida Glabrata.

## Introduction

An uncommon entity, emphysematous gastritis was first described by Fraenkel in 1889 [1]. It is a rare disease characterized by the presence of air within the wall of the stomach and diffuse gastric wall inflammation caused by gas-forming bacteria. Only 59 cases have been reported in the English language literature based on a review by Watson et al. and no guidelines are currently available for the management of emphysematous gastritis [2]. The most common bacteria implicated include Escherichia coli, Enterobacter species, Pseudomonas aeruginosa, Clostridium perfringens and Staphylococcus aureus [3]. The mortality rate is upwards of 60% with an increase to 75% on those requiring surgery [4]. Emphysematous gastritis caused by a fungal species is very rare. Herein we present such a case.

## Case presentation

**Figure 1:**
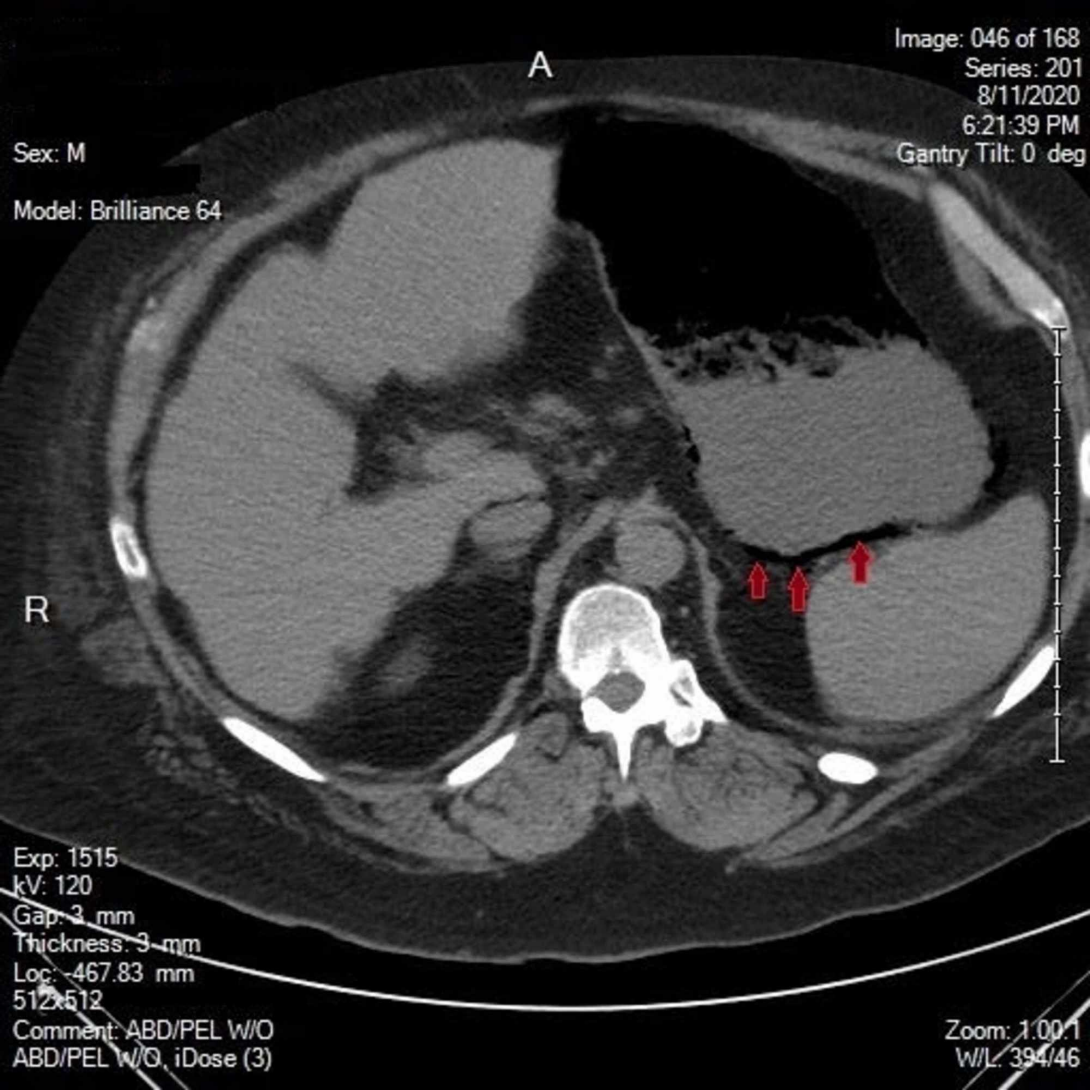
CT abdomen/pelvis demonstrating air within the wall of the stomach

**Figure 2:**
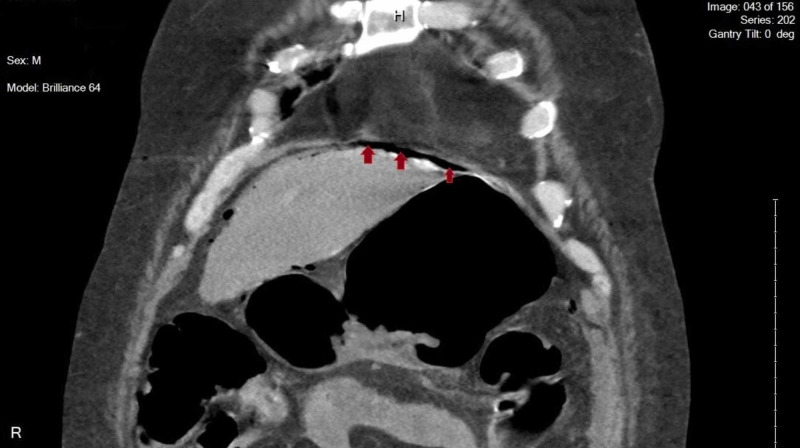
CT abdomen/pelvis demonstrating pneumoperitoneum

A 52-year-old Caucasian male with a significant past medical history of COPD, diabetes mellitus type 2, essential hypertension and chronic back pain presented to the emergency room with diffuse abdominal pain. The patient reported associated abdominal swelling with 3 episodes of nausea and vomiting in the past day. The patient also reports having felt lightheaded for the past couple weeks and reports falling 2 weeks ago. On arrival, the patient was afebrile with a blood pressure of 60/40, heart rate of 74, respiratory rate of 14 and saturating 98% on room air. Labs obtained were significant for a Na:130, Glucose: 115, BUN:46, Cr:4.3 (baseline:0.8), WBC:20.9, blood cultures grew gram + cocci. The patient denies drinking alcohol, smoking cigarettes or consuming anything corrosive. After not responding to fluids, a central line was placed and Norepinephrine started. CT of the abdomen demonstrated air within the wall of the stomach, portal veins and perigastric veins at which point surgery was consulted. After admission to the ICU, a follow-up EGD demonstrated Emphysematous gastritis secondary to gastric mucosal necrosis particularly in the proximal stomach just below the GE junction. The patient had already been placed on Piperacillin/Tazobactam and Metronidazole before admission to the ICU and after blood cultures came back positive for gram + cocci, Vancomycin was added. The following day the patient continued to complain of severe pain and follow up CT abdomen/pelvis demonstrated interval gastric perforation with mild free air within the upper abdomen and fluid within the posterior gastric wall representing gastric necrosis with perforation along with interval development of mild pneumoperitoneum (Figures 1, 2).

He was taken to surgery and underwent a subtotal gastrectomy with splenectomy due to becoming hypotensive during the procedure and the fear that attaching his distal esophagus to the jejunum at that point would result in poor perfusion. The decision was made to close him up and go back in a few days. Things became even more interesting when wound cultures taken during surgery came back positive for both yeast and streptococcus parasanguinis. Follow-up blood cultures were also positive for yeast. Micafungin was started at that point. He went back to surgery where his esophagus was found to have retracted into his chest, as well as his abdominal mesh being covered with pus. As much pus was cleared out as possible and was closed back up. In order to attach his esophagus to his jejunum, a thoracic surgical approach would need to be done for which the patient was not stable enough to perform. Peritoneal fluid cultures taken during the procedure grew yeast. With fungemia in his bloodstream, TPN had to be stopped. ID recommended stopping zosyn and adding meropenem. He remained intubated in the ICU where his family made the decision to compassionately extubate and make him comfort care. Culture wound results that had been sent for identification of the fungal species came back positive for Candida Glabrata.

## Discussion

**Figure 3:**
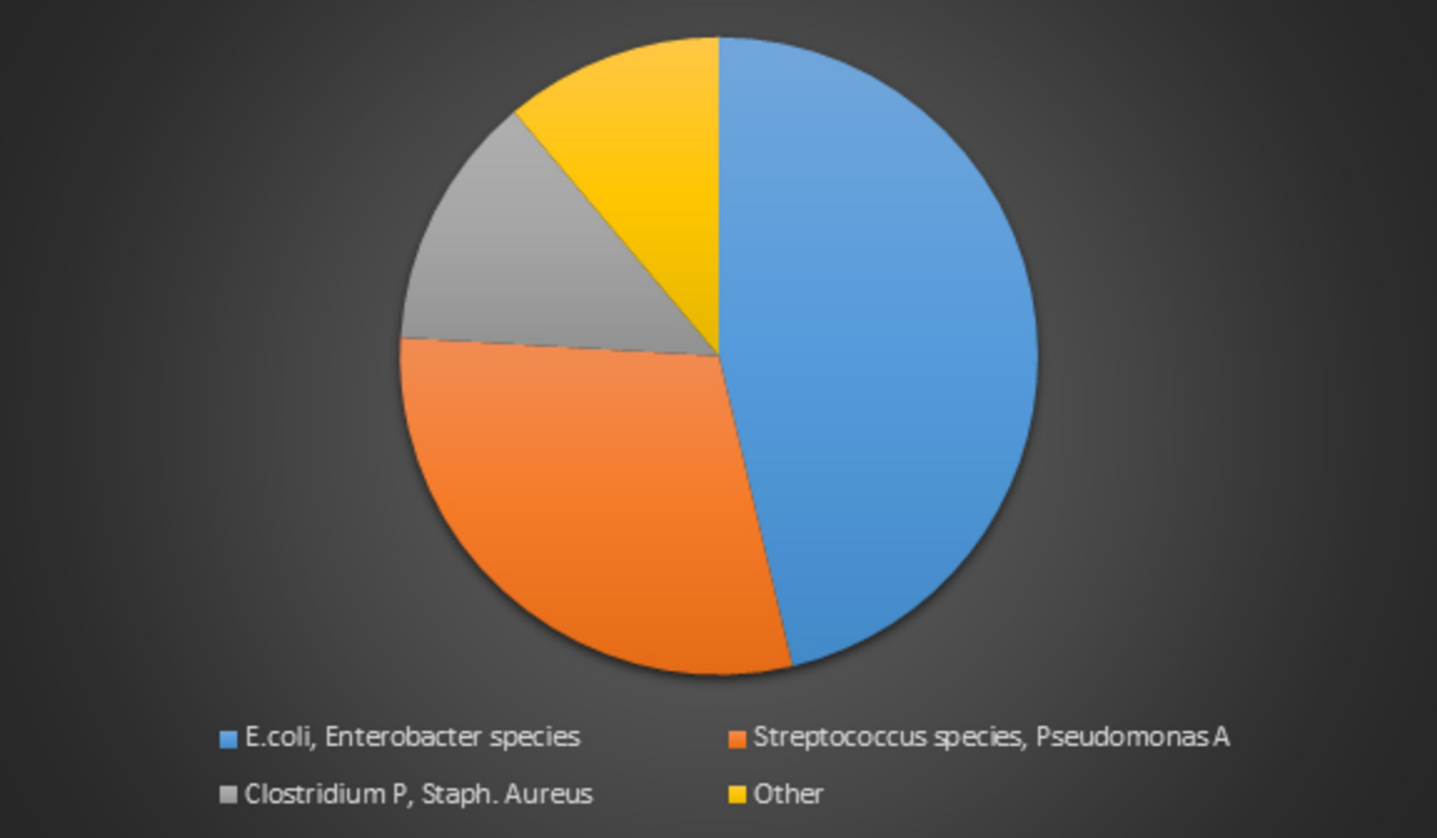
Graph representing different organisms implicated in Emphysematous gastritis based on available literature reviewed

Herein, we describe a case of Emphysematous gastritis caused by a fungal organism that occurred in the setting of pre-existing Diabetes Mellitus Type II with no other predisposing risk factors. A few terms used to describe gas in the wall of the stomach include gastric emphysema, pneumatosis intestinalis and emphysematous gastritis [5]. A distinguishing factor between these is that with gastric emphysema there is no associated infection as well as patients not presenting with acute abdomen and having an excellent prognosis even with no treatment. On the opposite side of the spectrum, emphysematous gastritis, caused by gas forming organisms, results in a necrotizing inflammation of the gastric wall. Emphysematous gastritis is a rare variant of phlegmonous gastritis caused by gas forming organisms and may arise from local spread through the mucosa or even hematogenous dissemination from a distant focus. The stomach is a very uncommon site of involvement because of its acidity and efficient mucosal barrier [6]. A review by Moosvi et al. of 27 cases from 1889 to 1990 showed that, in most patients, there was a prior insult to the mucosal barrier of the stomach, either by corrosives ingestion such as ammonia, acid (37%) or alcohol abuse (21%) [5]. A history of recent abdominal surgery and gastroenteritis was found in 15% each. Abdominal computed tomography, the best imaging modality to establish the diagnosis, reveals gastric wall thickening and intramural gas [7]. The gas in the wall is in the form of irregular mottled bubbles or spots, especially around the fundus and the greater curvature, and remains in place despite the position of the body or absorption through the gastric tube [8]. Organisms that have been cultured from stomach aspirates, blood, and peritoneal fluid include streptococci, E. coli, P. aeruginosa, Clostridium. perfringens, and S. aureus [9]. Among the previously reported cases, those associated with fungal infection were rare (Figure 3) [10,11].

We report a rare case of a 52-year-old male with associated DMII as the only predisposing risk factor developing Emphysematous gastritis complicated by perforation caused by a fungal organism. Diagnosed by clinical and radiological findings the patient went on to be made comfort care. We hope that this case will bring awareness to all medical providers in the hope of early diagnosis and intervention in order to increase the odds of survival.

## Conclusions

It is important to understand the etiology as well as the presenting symptoms of emphysematous gastritis in order to improve our approach and shorten the time to diagnosis. This will guide treatment therapy and lead to avoidance of complications requiring surgery which increases the mortality rate significantly. Although not a common entity, we must strive to identify it early in its course and improve patient outcomes.
